# Supplementary material for: Benefits of Playing at School: Filler Board Games Improve Visuospatial Memory and Mathematical Skills
Source: Brain Sci. 2024 Jun 26;14(7):642. doi: 10.3390/brainsci14070642 (PMC11274538; doi:10.3390/brainsci14070642)
Supplement: Supplementary file 1 [file brainsci-14-00642-s001.zip › brainsci-3059480-Supplementary material.pdf]

Table S1. Brief description of the games used in the Memory GTG and in the Math GTG and the primary/secondary theoretically activated outcomes.

Memory Filler Board Games Used in Memory GTG		
**Board Games**	**Primary Memory Outcomes**	**Secondary Mathematical Outcomes**
Alles Kanone! [58]	Visuospatial WM Updating	-
Alles Tomate! [59]	Visuospatial WM Updating	-
Chicken, Cha Cha Cha! [62]	Visuospatial STM	-
Out of Mine! [61]	Visuospatial STM and WM	Number Operations, Problem Solving
Spooky Stairs [60]	Visuospatial WM Updating	-

Alles Kanone! [58]: Seven themed cards are always face-up. One object card is positioned face-up, down from the themed card with which it is related. Once all of the players have seen the seven object cards, they are turned face-down. From the deck, one participant reveals a new object card. The first player to recall which element is on the face-down object card positioned down from the themed card with the same colour background is the person who will win the card. The topic of the game is about pirates. The player who gets the most cards becomes the winner.

Alles Tomate! [59]: This game follows the same rules as Alles Kanone!, but with a topic about farming.

Chicken, Cha Cha Cha! [62]: Four hen figures, with spaces at their back to place feathers, are positioned on one of the 24 egg-shaped (faced-up) tiles making a circle. In the middle of the circle, 12 octagons, each with a different chicken-related image, are positioned face-down. Each octagon image coincides with two egg-shaped tiles. To move his/her hen, the player has to find the correct combination of the octagon with one of the egg-shaped tiles. Once the octagon tile reveals its image, the tile is faced down another time. If one hen overtakes another, the one that overtakes steals the feather from the other one. The game ends when one player receives the four feathers. This person is the winner.

Out of Mine! [61]: Every player has a tunnel board randomly assigned, which has to be completed with different bidimensional pieces of different forms (each one representing a different type of mineral). All of the pieces fit together if they are properly combined. Moreover, each player has a card that indicates which pieces have to be used to win extra points. All players play at the same time. The topic of the game is about dwarfs in a mine. The player who gathered the most points after one work week (7 game rounds) wins the game.

Spooky Stairs [60]: It is a race to arrive at the top of the stairs. Each participant has a pawn of a specific colour. Rolling a 6-sided dice, each participant keeps climbing unless he/she rolls a "ghost". Then, a ghost piece, connected by a magnetic field to the pawns head, completely covers the participant's pawn. When all of the pawns are covered by the ghosts, all of the players must remember which the position of his/her ghost is. The game finishes when the first pawn reaches the top of the stairs. The player who belongs to this pawn is the winner.

Mathematical filler board games used in the Math GTG		
**Board Games**	**Primary Mathematical Outcomes**	**Secondary Memory Outcomes**
7ate9 [63]	Number Operations	-
Auf zack! [67]	Number Operations	Visuospatial STM

Numenko in a bag [64]	Number Operations	-
Pig 10 [65]	Number Operations	-
Shut the box [66]	Number Operations	-

7ate9 [63]: The 73 cards of the game are distributed for all of the players. One card is faced up at the centre of the table. Each card has a general number (from 1 to 10) and a smaller modifier in the corner (±1, ±2, or ±3). All of the players play at the same time, trying to add a card into the central pile (the one with the general number that applying the modifier score to causes the number of the central card to be modified). The person who first runs out of cards is the winner.

Auf zack! [67]: There are 44 cards from different families and a variable number of images, from 0 to 9. There are also 10 wooden pieces with a semi-circled form with a number written on it (it represents the solution). Play starts with a player turning two cards of a heap. If cards are from the same family, all players must calculate the sum of the two objects drawn on the cards and the first one to catch the wooden piece with the number of the sum, wins the card. The player with the maximum quantity of cards wins.

Numenko in a bag [64]: Some wooden pieces (with numbers from 0 to 9, operators or wildcards) are distributed through the participants and some others are placed in the middle of the table face-up. Players must combine the pieces to perform the maximum possible calculations, taking pieces from the middle if they need any one. When all of the players finish, all of the calculations receive points according to some rules. If there is a mistake or someone has used a wildcard, they receive a penalty of 10 points. The player who receives the lowest final score wins.

Pig 10 [65]*:* Players take 3 cards each from the 80 possible (all are numbered from 0 to 10). Every player, in his turn, has to put a card in the middle, taking into account the value of the card previously thrown, and steal another card from the heap. If the sum of the numbers from the stacked cards is equal to 10, the round finishes and the person who put in the last card receives all of the cards from the heap. If the sum is below 10, the turn is for the next player. If the sum is above 10, the person on the right of the player receives all of the cards of the heap. Some modifiers could be applied with special cards. The player with the greatest quantity of cards wins.

Shut the box [66]: The game's material includes one board with a plate in the centre, four groups of records numbered from 1 to 10 and two dices. In the beginning, all records are standing up. The player who starts throws the dices and sums the numbers of the results. The next step is to knock down the records, and the player can do this with the total sum or with a decomposition of it (i.e., if a player receives a 6, it can fall 6, 5 + 1, 4 + 2, 3 + 2 + 1). After three rounds, the player who has won the most times wins.
